# Outcomes of an Intervention Programme for People With Intellectual Disabilities and Behavioural Concerns Based on Emotional Development

**DOI:** 10.1111/jir.70008

**Published:** 2025-07-07

**Authors:** Allan Skelly, Jade Wigham, Mark Hudson

**Affiliations:** ^1^ Cumbria, Northumberland, Tyne and Wear NHS Foundation Trust Gateshead UK; ^2^ Mental Health and Clinical Neurosciences, School of Medicine University of Nottingham Nottingham UK

**Keywords:** attachment, emotional development, intellectual disability, intervention

## Abstract

**Background:**

This paper examines clinical outcomes of an approach to referrals for concerns about severe behavioural distress in people with intellectual disabilities (ID) that is based on the concepts of emotional development (EDev) and attachment. Due to research and clinical experiences suggesting that positive behaviour support (PBS) may have limited long‐term impact, an alternative approach is proposed, which focuses on relational factors and emotional skills already held by carers.

**Methodology:**

We report the process and outcomes of a brief standardised assessment using the Scale of Emotional Development–Short (SED‐S) which then informed an attachment‐based workshop. Outcomes were measured by way of the Health of the Nation Outcome Scales–Learning Disabilities (HoNOS‐LD) administered at (a) assessment, (b) following 1–2 intervention workshops and (c) at 6‐month follow‐up.

**Results:**

Significantly fewer sessions and hours of clinical time were required for the EDev intervention than the prior PBS interventions. Results demonstrated significant improvements from assessment to post‐intervention, which were maintained 6 months later. Almost half (48%) of the participants were referred while in unsettled accommodation arrangements.

**Conclusions:**

This study provides initial evidence supporting an approach that shows promise as an alternative to existing models of care based upon applied behaviour analysis. Future well‐controlled studies across multiple sites are needed, but if these findings are replicated, the priority for intervention may shift from reduction in behavioural risks to robust care relationships which meet the person's emotional needs.

## 1 Introduction

In this article, the initial outcomes of a brief intervention with professional care teams to address concerns about distressed or disturbed actions by people with intellectual disabilities (ID) are presented, based on the concepts of emotional development and attachment (the EDev pathway). Particularly with people re‐referred for the same concerns following prior intervention using positive behaviour support (PBS), this appears to have been effective in improving clinical outcomes in a clinical sample presenting to a community learning disabilities team within the National Health Service of the United Kingdom.

‘Challenging behaviour’ became a dominant idea within UK specialist learning disability services in the 1980s (Blunden and Allen 1987) as psychologists brought behaviour management techniques based on applied behaviour analysis (ABA) from institutional care into community settings. Intended as a liberating term that would allow services to alter the environment and address previously unmet needs (food, comfort and stimulation), it was envisaged that new learning would make ‘challenging behaviours’ redundant. Punitive and aversive methods, several of which would now be considered unethical, were dropped in favour of reward methodologies (Donnellan et al. 1988) and wedded into the values base of social role valorisation to create PBS, clearly set out by Gore et al. (2013). The accepted definition defines challenging behaviour as that which risks serious, irreversible physical harm to the self or to others, and/or which risks the person's liberty being restricted (see British Psychological Society/Royal College of Psychiatrists 2016).

PBS is an explicitly evidence‐based approach, which is widely used in specialist ID services as a full model of care, not merely an intervention. However, the evidence for effectiveness is mixed, with significant gaps. A recent large‐scale study that attempted a multisite randomised controlled trial of PBS did not detect any superiority over a treatment‐as‐usual control condition, although the outcomes may have been affected by poor implementation of PBS plans (Hassiotis et al. 2018). On a smaller scale, McGill et al. (2018) have demonstrated that by incorporating coaching and monthly review of social care practice within PBS interventions, reductions in the rate of challenging behaviour can be achieved and maintained at 12‐ to 18‐month follow‐up. Similarly, Hassiotis et al. (2009) randomised 63 adults referred for challenging behaviour to either ‘standard care’ (medication, nursing and adaptive skills training) or to a specialised intervention based on PBS, provided by qualified trainers in ABA, plus standard care. Significantly greater improvements were found in the enhanced care group, which were maintained at a 2‐year follow‐up, although this study was limited by poor description of the PBS intervention (Hassiotis et al. 2011).

A recent systematic review by Konstantinidou et al. (2023) identified nine relevant outcome studies of PBS for children and adults with disabilities, where the intention was to evaluate the impact of organisational behaviour management systems on staff and service user behaviour. Certain studies (Dench 2005; MacDonald et al. 2018; McGill et al. 2018) showed statistically significant reductions in challenging behaviour recordings in the short term, but not increased quality of life (QoL), and only two studies were described as methodologically strong. This review was limited in scope; however, it focused on the delivery of training to non‐specialists, which may affect the estimates of PBS outcomes. A similar finding was also reported by Simler (2019) in a systematic review of PBS interventions for people with ID, which included 15 studies, of which a majority (73%) were found to be low quality. Overall, there is preliminary evidence of PBS interventions leading to short‐term reductions in challenging behaviours that have been maintained in an isolated number of studies. However, Simler (2019) concluded that, as it stands, PBS cannot claim to enhance QoL, teach adaptive skills or reduce the use of restrictive practices. Improvements in intervention fidelity, social care involvement and the use of appropriately qualified instructors are needed to further evidence the effectiveness of PBS in the longer term and across a range of outcomes.

The field of emotional development offers a conceptual and practical alternative to PBS to understand concerns about behaviour. It is not widely used in UK specialist clinical teams, despite a strong empirical basis and explanatory potential (e.g., Hermann et al. 2024; Sappok et al. 2020, 2022; Kasari et al. 2012). Of the several models of emotional development, Došen's phase‐based model (Sappok et al. 2021) has been most widely implemented in ID settings across Europe. This model proposes that emotional development progresses through five phases (later extended to six, to include people with borderline intellectual functioning):

1. Adaptation (0–6 months): associated with sensory integration.
2. Socialisation (6–18 months): associated with the development of object permanence and attachment relationships.
3. First individuation (18 months to 3 years): exploration from a secure base; developing a sense of self and autonomy
4. Identification (3–7 years): development of theory of mind (mentalisation), improving peer relations, reduced aggression, and compliance in formal educational settings.
5. Reality awareness (8–12 years): reflective thinking, logical reasoning, moral action.

Emotional development typically occurs in tandem with social and cognitive development, although emotional experience can be considered a primary driver of subsequent mental abilities (Greenspan 1997). A range of factors may lead to a desynchrony to occur, where emotional development is delayed in relation to social and cognitive development, including the presence of ID, other neurodevelopmental conditions, attachment difficulties or significant childhood trauma. This might lead to overestimation, poor care planning and associated behavioural distress (Došen 2005). Using the emotional development approach, mental health difficulties and behaviours of concern can be understood and often normalised in their developmental context (Došen and De Groef 2015; Hermann et al. 2022).

A report by the British Psychological Society (2017) found that early attachment difficulties are linked to a broad range of negative health outcomes in people with ID. These include impairments in the development of empathy and self‐reflection in children, social difficulties at school, increased likelihood of neurodivergent diagnoses, depression, diagnosis of compulsive behaviours, substance misuse and even psychosis. Although this document outlined several promising clinical interventions with good preliminary evidence, attachment interventions are not mentioned within UK policy or practice guidelines for people with ID. This is in stark contrast to children in the care system, who are the focus of a specific guideline promoting permanent attachments within family‐style care, over genetic explanations, medication and institutional care practices (National Institute for Health and Care Excellence 2015, 2016).

In a systematic review of attachment and ID, Hamadi and Fletcher (2021) found that ID is a risk factor for attachment difficulties, either through substantially heightened exposure to childhood maltreatment (Fang et al. 2022) or the difficulties caused by the disability in emotional expression, connection and delay or difference in how emotional development can progress. Attachment theory can be used successfully in clinical work with people with ID (Fletcher et al. 2016), and so we sought to progress this.

In our service in North‐East England, a referral that details concerns about the risks of certain behaviours/actions by the person with ID is considered primarily in terms of how well the person's emotional development is understood. A key question is whether the person's carers have a clear idea of the person's emotional phase, so that they can make realistic judgements about the purpose and intentions behind any actions that may challenge their ability to support and care for the person. There is usually some degree of inaccuracy in the judgements made, particularly over‐estimation. When the dominant phase of emotional development is found, we can then educate the care team (and/or family) about how to develop the relationship with the person so that the connection becomes more permanent, via (1) reinforcing the primacy of *physical and emotional safety*, (2) *exploration at the right level* for the particular phase, with the right amount of proximity and availability for support, (3) *engagement with activities and tasks* with the right amount of expected co‐completion or self‐occupation and (4) comforting and reassuring appropriate for the identified phase. It is very important to determine if the person has passed the ‘theory of mind threshold’ (Phase 4 and above) as this will determine if self‐regulation, empathy, self‐occupation and a range of other emotional capacities can be expected.

**FIGURE 1:**
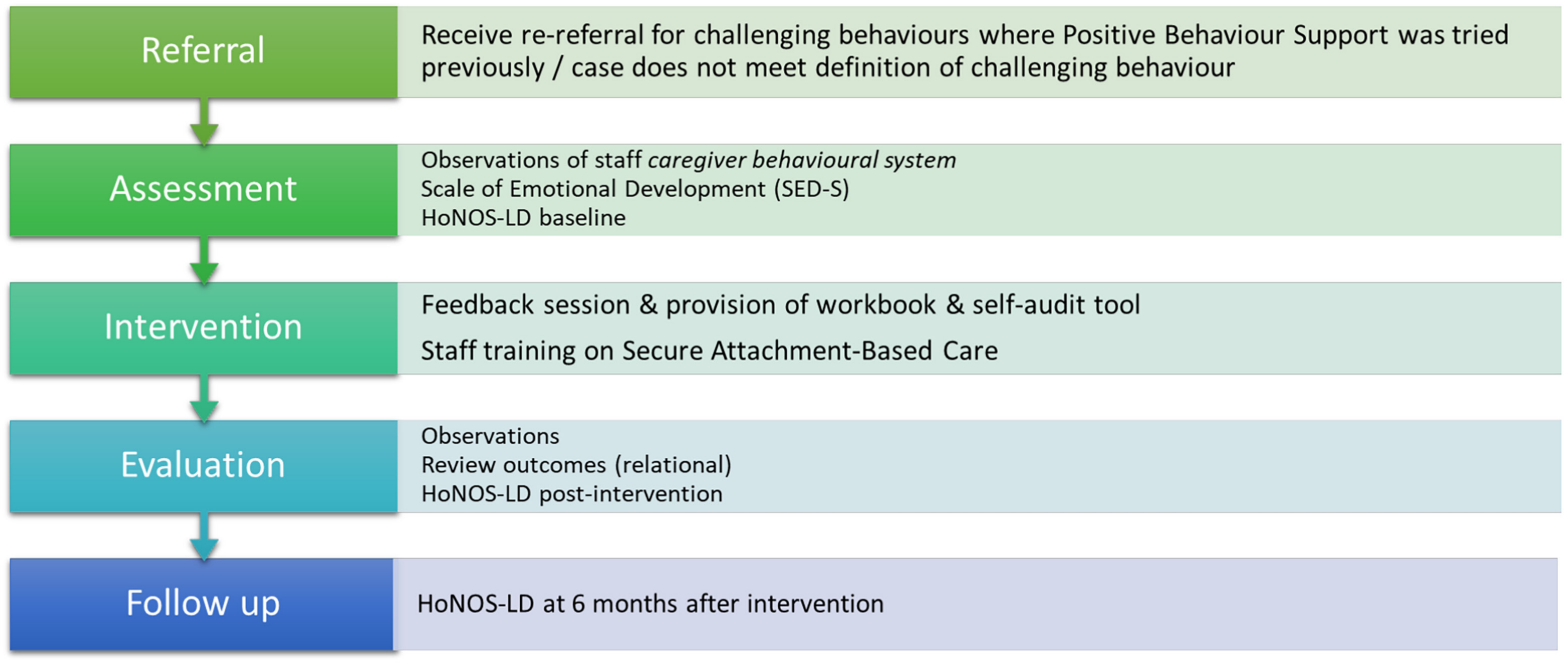
Emotional development pathway for referrals mentioning behaviour difficulties in a community learning disabilities team.

In the current study, a PBS pathway was previously followed for the majority of cases (29/35; 82%) and not found to be successful in resolving the risks associated with the behaviours of concern. A small number of cases (6; 18%) were ‘diverted’ to the EDev pathway when they were referred to the PBS pathway, but the problems did not meet definition of challenging behaviour (i.e., not sufficient to cause significant harm or restrictions). The EDev pathway for the team is shown in Figure 1.

This paper presents an initial examination of outcomes of our EDev pathway by comparing an assessment phase baseline (1) to immediately following the intervention (2) and then at 6‐month follow‐up (3). From prior clinical experience and measuring outcomes in individual cases, we hypothesised that a grouped caseload would demonstrate positive outcomes over time, though we were less sure if this would be the case at follow‐up. The pathway was prospectively designed with mandatory outcomes measurement in place to enable data analysis and reporting of the results.

## 2 Method

### 2.1 Participants

Twenty‐nine people with an ID who were re‐referred to a community learning disabilities team for difficulties which met the definition of challenging behaviour (BPS/RCP 2016) were seen on the emotional development pathway. Cases were not subject to a waiting list as the service had a minimal wait. Six people referred for the first time whose actions did not meet the definition of challenging behaviour, but who were considered to potentially benefit from the pathway, were included in the study. They had not received a prior multidisciplinary behaviour support intervention but had instead received a functional assessment and a report containing advice based on this assessment. The EDev pathway is relatively unobtrusive, being based on indirect assessment, and no clients were excluded following the offer of the assessment. We had intended that if an external agency or clinician working elsewhere in the service had offered further PBS intervention, then we would exclude that case from the data analysis, but this did not occur.

Fifteen participants (43%) were adult women, and 20 (57%) were adult men. They were mostly younger than 40 years (*M* = 37.17, Mdn = 30, SD = 16.45, range = 19–74). During the intervention period, 15 cases (43%) experienced a change to medication to address their mood or behaviour (*M* = 1.14 changes, Mdn = 0, SD = 1.93, range 0–8). Eighteen (51%) cases were referred following physical aggression, all of whom were also verbally aggressive. Twelve cases (34%) were referred following self‐injury, and of these, seven (20%) demonstrated both aggression and self‐injury. There were nine cases (26%) where only verbal aggression was observed; however, all of these were cases where historically, physical aggression had occurred, and there was concern that it could occur again. One case (3%) was referred following arson, and one case was referred for excessive passivity which led to severe self‐neglect. Six cases (17%) were supported in the family home by carers who visited daily (care at home), 12 (34%) lived in their own home with < 24‐h care, five (14%) lived in multiple occupancy accommodation with 24‐h staffing (though shared) and 12 (34%) were living in varying forms of emergency placement, one of which was a hospital. We did not record the exact staffing ratios within the care plans. Some 17 of the 35 (48%) participants were in unsettled accommodation arrangements, that is, there was official notification that they were due to move but had not yet done so.

To identify levels of disability, we utilised Item 6 (cognitive understanding) of the HoNOS‐LD measurement at baseline to estimate level of ID. Ratings suggested that nine people (26%) had no difficulty understanding the first language of their culture, nine (26%) were able to understand groups of words, short phrases and instructions from familiar people, 16 people (46%) could only understand individual signs/gestures or single words, and 1 person (3%) was only able to recognise that people were communicating with them with no apparent meaningful understanding. No participants achieved the most severely impaired rating (no apparent understanding or response to communication).

The Scale of Emotional Development–Short (SED‐S) (described below) yields an overall phase of emotional development based on the median phase of the eight domains. Of the 35 participants, 3 (9%) had reached adaptation (0–6 months); 4 (11%) had reached socialisation (6–18 months); 23 (66%) had reached first individuation (1½ to 3 years); and the remaining 5 (14%) had reached identification (3–7 years). None had reached reality awareness (8–12 years).

### 2.2 Measures

#### 2.2.1 The Scale of Emotional Development–Short (SED‐S)

The SED‐S is a 200‐item binary choice measure which determines an individual's level of emotional development as defined by Došen's phase model. The SED‐S is comprised of eight domains, each of which includes five items in each level of emotional development. The eight domains include (1) relating to his or her own body, (2) relating to significant others, (3) dealing with change/object permanence, (4) differentiating emotions, (5) relating to peers, (6) engaging with the material world, (7) communicating with others and (8) regulating affect (Sappok et al. 2016, 2022). The SED‐S provides the current level of ED on domain and overall level in children and adults with an ID. It was administered at baseline by a higher assistant psychologist under the supervision of an HCPC accredited clinical psychologist via a semi‐structured interview with close caregivers and took about 30–60 min to complete. In 160 typically developed children, the SED‐S showed a weighted kappa of 0.95 and 81% exact agreement between the chronological age of the children and the emotional reference age as assessed with the scale (Sappok et al. 2019). The SED‐S shows high internal consistency; Cronbach's α was 0.99 in typically developed children; 0.94 in children with an ID (Sterkenburg et al. 2021); 0.92 in adults with ID (Meinecke et al. 2024).

#### 2.2.2 HoNOS‐LD

An adapted version of the Health of the National Outcome Scales—Learning Disability (Roy et al. 2002) was administered with carers who have known the person for at least 2 months at (a) baseline, (b) within 2 weeks of the intervention and (c) at 6 month follow‐up. The HoNOS‐LD was proposed as a generic measure of outcome in specialist learning disability services and has been shown to have good inter‐rater reliability and validity characteristics (Painter et al. 2023). As well as a total score based on the 22 items, four factor scores developed by Skelly and D'Antonio (2008) were produced based on items that pertain to:

- Cognitive and communicative competence (CCC; not expected to change significantly after intervention);
- Disturbance of behaviour, mood and relationships (BMR);
- Loss of adaptive functioning (LAF);
- Severe agitation (SA) or internal dysregulation.

The statistical package PSPP (GRU freeware) was used for the purposes of inferential analyses. The HoNOS‐LD data for all five HoNOS‐LD scores (Total, CCC, BMR, LAF and SA) were normally distributed in most instances (skew and kurtosis < 1.0), with the exceptions of the BMR follow‐up scores (kurtosis = 2.48), the CCC post‐intervention scores (kurtosis = −1.19) and the baseline and follow‐up scores for SA (kurtosis = 4.15 and 9.33 respectively). SA scores were also skewed at baseline (1.38) and follow‐up (2.57). As the assumptions for parametric statistics were violated in these instances, we utilised a series of conservative Friedman tests.

We created a continuous variable representing the change in HoNOS‐LD scores from baseline to intervention completion that was normally distributed (skew = −0.08, kurtosis = 0.20). This enabled us to estimate associations between therapeutic change and age, number of hours in the PBS pathway, number of hours in the EDev pathway, additional psychological therapy sessions, number of medication changes made during the EDev pathway, gender and level of disability.

### 2.3 Intervention

The intervention predominantly consisted of a single feedback session for carers, all of whom were professional care staff, with placements commissioned by the local authority or NHS integrated care board. The intervention comprised the following:

- an introductory video about attachment theory from the public domain *Sprouts* organisation,
- a review of the impact of childhood adversity and attachment difficulties on mental health problems,
- explanation of the person's dominant phase of emotional development demonstrated by the SED‐S assessment, outlining expectations of that phase.

Demonstration of how to apply attachment‐based caregiving principles according to the specified phase, utilising a circular model of attachment‐based caregiving adjusted to the dominant phase of emotional development for each case. This is based on four circular tasks headed (1) providing physical and emotional safety; (2) promoting exploration; (3) promoting engagement in joint activities; and (4) providing comfort and reassurance in response to distress or anxiety. An additional slide gave specific subpoints about proximity and warmth, the amount of direction required in order to explore effectively, expectations of the extent to which tasks are suggested, prompted or done together, and co‐regulation of distress where the person struggles to self‐regulate.

- Provision of a workbook containing a diagrammatic reference sheet for the four‐phase model at each level of emotional development, brief guidance notes and a self‐assessment tool to be used at a service level to gain organisational feedback.

The most common phase to present was phase three (first individuation), which is normally observed in typically developing children between the ages of 1.5 and 3 years. Much of the feedback to the care teams involved lowering expectations of mentalisation (theory of mind), the ability to self‐occupy, chronological age‐related activities and peer‐based activity, towards a model of care where staff provide structure and limited choices that are not too overwhelming or confusing.

The model of secure attachment caregiving presented here is relatively clear once the level of emotional development is known. Similar models utilising this secure base–safe haven approach have been applied in the area of child development (e.g., circle of security: Powell et al. 2014) and ID research (e.g., De Schipper and Schuengel 2008). The ‘4 tasks’ of emotionally secure caregiving is essentially a model of the warm, attentive, attuned and responsive parent associated with secure attachment in child development. The model is adjusted so that each task is appropriately differentiated according to the dominant emotional phase the person has reached.

Further non‐directive support sessions (with no limit on frequency) or a repeat of the workshop were on offer for all the receiving care teams, but within the study period, none were requested. Some informal feedback has been given in further multidisciplinary meetings after the conclusion of the study, but the generally positive feedback is outside the scope of the study period and did not involve further formal measurement.

## 3 Results

### 3.1 Phases of Emotional Development

The dominant phases of emotional development were distributed as follows: 3 cases (8%) at Phase 1 (adaptation); 4 cases (11%) at Phase 2 (socialisation); 23 cases (66%) at Phase 3 (first individuation); 5 cases (14%) at Phase 4 (identification). No cases were identified at Phase 5 (reality orientation) or Phase 6 (social individuation).

### 3.2 Differences in Input Levels

The number of formal training sessions involved in the EDev intervention (*M* = 0.97, Mdn = 1, SD = 0.51, range 1–3) was fewer than the number of training sessions for PBS (*M* = 2.40, Mdn = 2, SD = 1.97, range 0–8). Even including the six cases with zero PBS training sessions, this result was highly significant (Wilcoxon test: *Z* = −4.02, *p* < 0.001). The average EDev intervention took approximately 90 min for the EDev training (*M* = 1.49 h, Mdn = 1.5, SD = 1.09, range 0.45–6) versus more than 6 h for PBS (*M* = 6.26, Mdn = 4, SD = 6.21, range 0–26). Again, this was highly significant (*Z* = −4.51, *p* < 0.001). However, lower levels of input would mean little unless this was also associated with positive clinical outcomes. Where there was more than one workshop, this was due to not all staff being available for the first workshop. Workshops were delivered to at least two members of the care team, up to a maximum attendance in the training room of 12, but the numbers of carers in attendance were not counted each time.

### 3.3 Main Effects of Intervention From Baseline to Follow‐Up on Clinical Outcome

No cases dropped out of the intervention, meaning there was 100% adherence to workshop attendance. Given that we used five outcome scores across three conditions, the Bonferroni correction was used to control for Type 1 error. Friedman tests showed significant decreases for Total HoNOS‐LD score from baseline to intervention (mean ranks for baseline = 2.83, post‐intervention = 1.73, follow‐up = 1.44; test statistic = 38.86 [2df], corrected *p* < 0.001). Unexpected improvements in communication and cognitive competence (CCC) were also found (mean ranks for baseline = 2.29, post‐intervention = 2.01, follow‐up = 1.70; test statistic = 12.95 [2df], corrected *p* = 0.002). Behaviour–mood–relationships (BMR) scores showed marked improvements post‐intervention (mean ranks for baseline = 2.74, post‐intervention = 1.76, follow‐up = 1.50; test statistic = 32.20 [2df], corrected *p* < 0.001). The pattern of improvement was repeated for LAF scores (mean ranks for baseline = 2.54, post‐intervention = 1.80, follow‐up = 1.66; test statistic = 20.15 [2df], corrected *p* < 0.001). Finally, SA was also shown to significantly improve (mean ranks for baseline = 2.39, post‐intervention = 1.86, follow‐up = 1.76; test statistic = 14.15 [2df], corrected *p* = 0.001). Where the factor scores were normally distributed (total scores, BMR, LAF), it was possible to consider the size of the pre–post effect from baseline to post‐intervention: (total score; *Hedges' g* = 0.89, 95% CI 0.52–1.29, BMR; *Hedges' g* = 1.06, 95% CI = 0.62–1.53, LAF; *Hedges' g* = 0.49, 95% CI 0.19–0.80).

### 3.4 Difference Between Completion of Intervention and Follow‐Up

**FIGURE 2:**
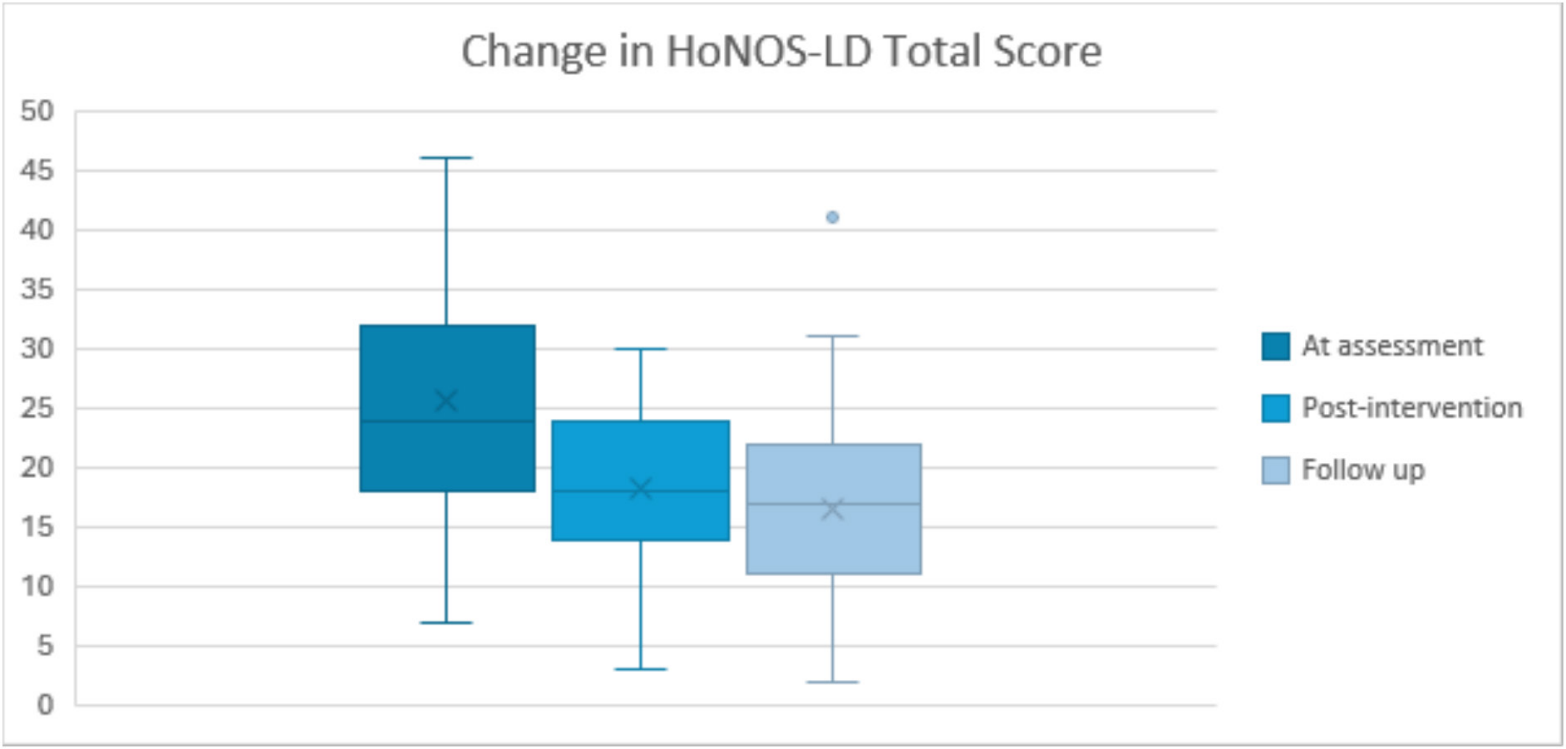
Box and whisker plot for HoNOS total score at baseline, post‐intervention and follow‐up.

For comparison, although scores continued to fall from post‐intervention to follow‐up, the effect sizes were smaller, suggesting most change happened between baseline and the completion of the intervention (total score; *Hedges' g* = 0.21, 95% CI −0.06 to 0.48, BMR; *Hedges' g* = 0.11, 95% CI −0.23 to 0.45, LAF; *Hedges' g* = 0.18, 95% CI −0.14 to 0.53). To further examine the possibility of a ‘sleeper effect’ on the total HoNOS‐LD scores, an additional Wilcoxon sign‐rank test excluding the baseline scores suggested that no significant effect was present (*Z* = −1.42, *p* = 0.16). However, a lack of worsening in the scores implies maintenance of improvements. Figure 2 demonstrates the change across time for the HoNOS‐LD total scores.

### 3.5 Potential Influence of Extraneous and Demographic Factors on Change

Spearman rank‐order correlations of client age, hours of intervention in the PBS pathway, hours of intervention in the EDev pathway, additional psychological therapy hours and medication changes did not find any significant associations with pre–post change in total HoNOS‐LD scores (rho = −0.09,‐0.20, −0.09, 0.05 and 0.03, respectively). Gender was not associated with therapeutic change on the total HoNOS‐LD score (*F*[1,34] = 1.68, *p* = 0.19), nor was the person's level of disability, as represented by the cognitive understanding item of the HoNOS‐LD (item 6: *F*[2,32] = 0.41, ns).

However, people who had remained within an active PBS pathway for longer tended to experience more medication changes during the EDev intervention period (rho = 0.37, *p* < 0.05). Taken together, these results suggest more confidence that the change observed following EDev intervention is not affected by the quantity of input (formerly or currently), client demographics or level of disability.

## 4 Discussion

These findings offer some preliminary evidence that a brief intervention utilising emotional development and attachment theory was associated with positive outcomes in a clinical sample of people with ID, who were referred following concerns about their recent actions. Improvements were maintained at follow‐up. Most of these cases were associated with Phases 1–3, where the person can be expected to lack the development of certain emotional capacities that allow for formal learning, reasoning, other‐centredness and ultimately emotional self‐regulation. Although mechanisms of change were not explored in this study, McGregor (2022) found that the administration of the SED‐S with care teams leads to a ‘lightbulb’ moment and significant lowering of expectations in terms of emotional independence. This may support more empathic, intensive and often structured support from the caregiver.

The outcome of the large‐scale randomised controlled trial of PBS by Hassiotis et al. (2018) led to the authors suggesting that alternative approaches are needed to support vulnerable people with ID whose actions cause concern. It is acknowledged that the non‐significant results of the RCT may be attributable to poor fidelity to the model, as the trainers did not engage sufficiently with the required skills development (Allen et al. 2018). We have found that the EDev intervention is very accessible and does not require a lot of technical learning. It is encouraging that in most cases there was no resurgence in the original behaviour (c.f. Pritchard et al. 2014), though of course one small‐scale study with a single point of follow‐up is insufficient to exclude resurgence as a significant issue.

This study has several limitations. A control group, such as treatment‐as‐usual or PBS refresher, would be an important design improvement. The sample size was small and from one locality in Northern England, where diversity is lower than the UK average (> 90% White British compared with 76% for the UK as a whole), limiting any generalisability of the findings. Exact staffing ratios were not recorded, which could potentially affect the outcomes of the intervention. We did not observe and/or code changes in caregiver attachment behaviours using observation (except unsystematically in some cases as a clinical action), and so this presents a gap in our understanding between the workshops and the outcomes. Further research should enquire about changes to the carer's thoughts, feelings and actions after a workshop intervention. Improvements may have been entirely due to the impact of the SED‐S assessment alone, spontaneous improvement or another factor.

As with psychological intervention research generally, there is some potential for allegiance effects, as well as a lack of heterogeneity in the intervention team (the same clinical psychologist was present in all developmental feedback sessions). It could therefore be argued that effectiveness was based on the working alliances and therapist characteristics of that individual, which may not be generalisable to other practitioners. These limitations can largely be addressed by a multisite trial. However, as with all psychological therapies, complete blinding to the treatment offered is not possible.

An interesting additional finding is that higher amounts of prior PBS practitioner input were associated with more medication changes during the EDev study, although the median number of such changes was zero. This implies an association between more intervention by a PBS practitioner and more involvement by a psychiatrist, as teams co‐ordinate their responses to match how needs spike or recede. However, causality between PBS intervention leading to more medication changes or vice versa appears unlikely.

We noted a high number of cases where the person's living situation was uncertain during the study (48% of the sample). This presents a challenge to an attachment‐based intervention in situations where staff turnover may be high, but this was not measured in the current study, which is a limitation. Informally, we did notice that there may have been a more negative or even rejecting attitude in care teams who were in the process of handing over to a new team, which may be due to prior over‐estimation or carer fatigue. We would argue that even in short‐term placements, emotional attunement from caregivers is important to develop safety with the client. Clinical experience suggests that many services lack emphasis on carer‐client relationships, focusing instead on behaviour counts and risk reduction. Multiple moves can lead to feelings of loss and grief and reinforce attachment insecurity.

This is the first study of its kind that considers working with emotional development and attachment in response to concerns raised about the impact of behaviour. Referrals in specialist ID services commonly describe challenging behaviours without reference to early history, trauma, attachment difficulties or emotional development. This may predispose interventions towards medications, activity planning and reinforcement programmes that could be delivered by multiple, interchangeable staff who lack significant emotional connection to the client. Instead, we propose that ideally, care arrangements will ensure each person is afforded a clear understanding of emotional development, imparted to a small number of staff with a warm, committed and attuned bond to the person. After all, the best care must feel like being loved by significant others (Shimmens and Skelly 2023).

## Ethics Statement

The study was conducted as part of routine service evaluation, and ethical approval was not required; however, it was registered with the Cumbria, Northumberland Tyne and Wear NHS Foundation Trust. The study was carried out in accordance with the British Psychological Society's (2021) Code of Human Research Ethics.

## Conflicts of Interest

The authors declare no conflicts of interest.

## Data Availability

The data that support the findings of this study are available on request from the corresponding author. The data are not publicly available due to privacy or ethical restrictions.
